# Supplementary figures and images for: Proof-of-Concept Analysis of B Cell Receptor Repertoire in COVID-19 Patients Undergoing ECMO by Single-Cell V(D)J and Gene Expression Sequencing
Source: Curr Issues Mol Biol. 2023 Feb 9;45(2):1471–82. doi: 10.3390/cimb45020095 (PMC9955795; doi:10.3390/cimb45020095)

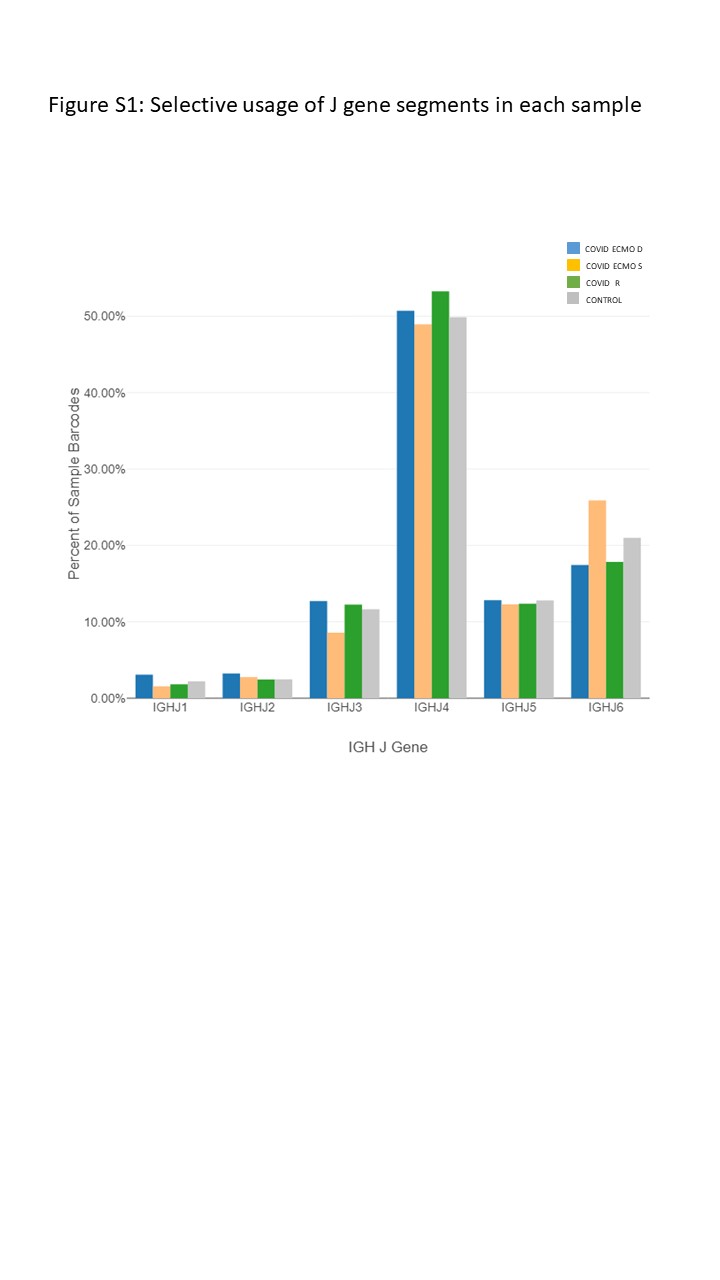

Supplement: Supplementary file 1 [file cimb-45-00095-s001.zip › supplementary Figures/S1.jpg]

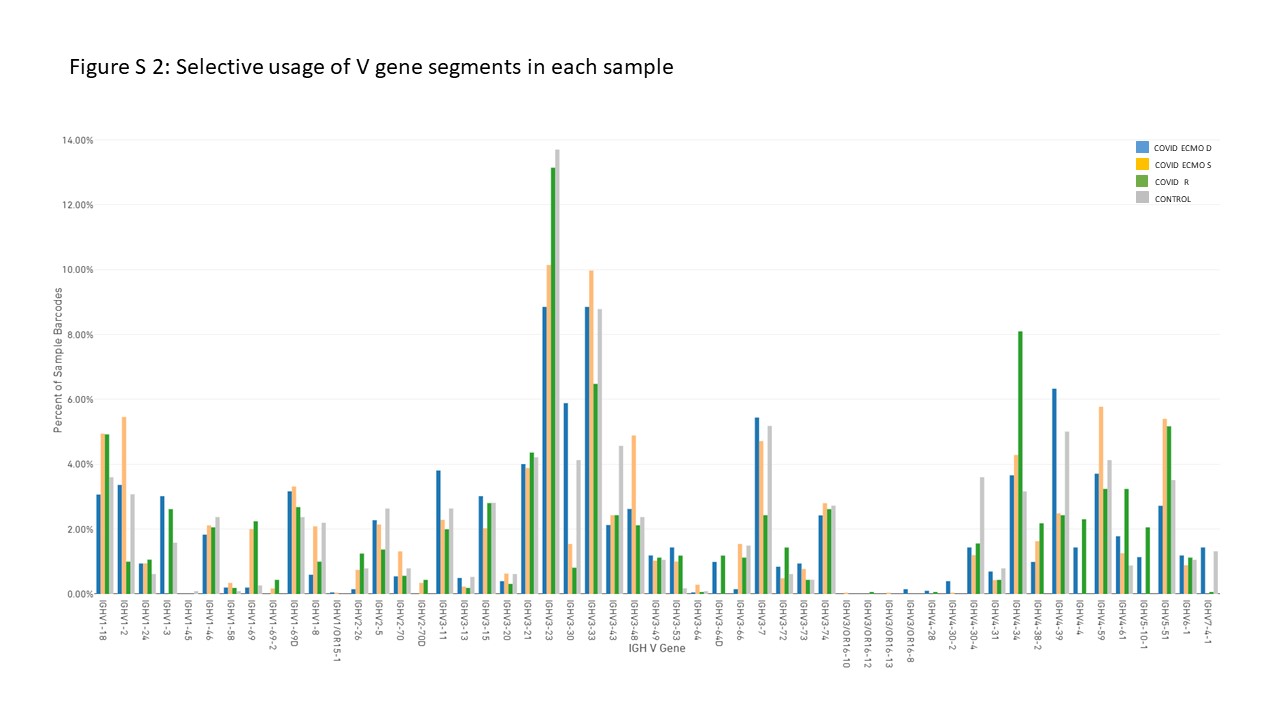

Supplement: Supplementary file 1 [file cimb-45-00095-s001.zip › supplementary Figures/S2.jpg]

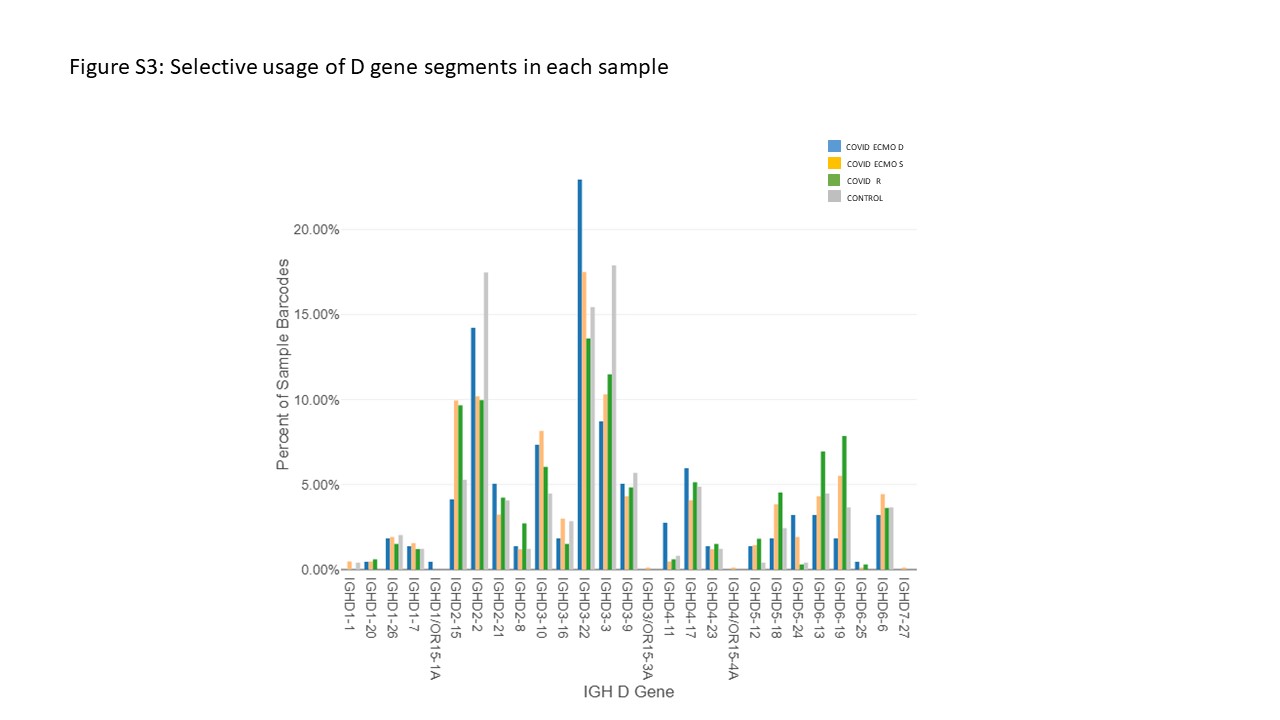

Supplement: Supplementary file 1 [file cimb-45-00095-s001.zip › supplementary Figures/S3.jpg]
